# Dynamic Changes of Plasma Metabolome in Response to Severe Feed Restriction in Pregnant Ewes

**DOI:** 10.3390/metabo9060112

**Published:** 2019-06-10

**Authors:** Changzheng Guo, Yanfeng Xue, Hossam-eldin Seddik, Yuyang Yin, Fan Hu, Shengyong Mao

**Affiliations:** 1 Laboratory of Gastrointestinal Microbiology, College of Animal Science and Technology, Nanjing Agricultural University, Nanjing 210095, China; guocz312@163.com (C.G.); xueyanfeng1990@163.com (Y.X.); 2016205033@njau.edu.cn (H.-e.S.); HFjuly@163.com (F.H.); 2 Jiangsu Key Laboratory of Gastrointestinal Nutrition and Animal Health, Nanjing Agricultural University, Nanjing 210095, China; 3 National Experimental Teaching Demonstration Center of Animal Science, Nanjing Agricultural University, Nanjing 210095, China; 4 National Center for International Research on Animal Gut Nutrition, Nanjing Agricultural University, Nanjing 210095, China; 5 Joint International Research Laboratory of Animal Health and Food Safety, Nanjing Agricultural University, Nanjing 210095, China; 6 Huzhou Academy of Agricultural Sciences, Huzhou 313000, China; yinyuyang@163.com

**Keywords:** dynamic changes, HPLC-MS, severe feed restriction, pregnant ewes, ketone bodies

## Abstract

Maternal metabolic disorders in ewes induced by energy deficiency have a detrimental effect on the maternal health and lambs. However, the dynamic processes of metabolic disorders are unknown. Therefore, this study attempted to explore the dynamic changes of maternal metabolism based on metabolomics approach during energy deficiency in pregnant ewes. Twenty pregnant Hu sheep were fed a basic diet or a 70% restricted basic diet. The HPLC-MS platform was applied to identify blood metabolites. Principal component analysis of blood samples based on their metabolic profile showed that blood samples of feed restriction group differed after the treatment. In particular, when comparing both groups, there were 120, 129, and 114 differential metabolites at day 5, day 10, and day 114 between the two groups, respectively. Enrichment analysis results showed that four metabolic pathways (glycerophospholipid metabolism, linoleic acid metabolism, arginine and proline metabolism, and aminoacyl-tRNA biosynthesis) at day 5, four metabolic pathways (aminoacyl-tRNA biosynthesis, aminoacyl-tRNA biosynthesis, glycerophospholipid metabolism, and citrate cycle) at day 10, and nine metabolic pathways (aminoacyl-tRNA biosynthesis, synthesis and degradation of ketone bodies, glycerophospholipid metabolism, butanoate metabolism, linoleic acid metabolism, citrate cycle, alanine, aspartate and glutamate metabolism, valine, leucine and isoleucine biosynthesis, and arginine and proline metabolism) at day 15 were significantly enriched between the two groups. These findings revealed temporal changes of metabolic disorders in pregnant ewes caused by severe feed restriction, which may provide insights into mitigation measures.

## 1. Introduction

Previous studies show that fatty acids stored in adipose tissue in the form of triglycerides are released and transported into mitochondria for β-oxidation to produce acetyl-CoA [1,2]. However, acetyl-CoA produced by fatty acids β-oxidation produces a large amount of ketone bodies which can be cytotoxic at high concentrations [3]. Previous results showed that severe feed restriction (FR) significantly reduced blood glucose levels and increased blood β-hydroxybutyric acid (BHBA) levels in the ewes [4]. In addition, liver metabolic profiling results suggested that severe FR caused disorders of liver lipid metabolism and impaired liver metabolic function [5]. Given that identification of the early steps in the development of fatty liver is important for development and use of early indicators of fatty liver during malnutrition, the understanding of the potential biomarkers in metabolic disorders caused by insufficient energy is of great significance, especially to pregnant animals [3]. In addition, previous studies mainly focused on the effects of maternal energy deficiency on offspring [6,7], but there are few studies focused on the mechanism of maternal body metabolic disorders and pathogenesis.

Animal models have been used to fill the knowledge gap between maternal diet and adult disease with clear mechanistic details [8,9]. A previous study revealed that it is easy to cause negative energy balance in polytocous sheep during pregnancy that can cause pregnancy toxemia [10]. Previous studies have successfully investigated the relationship between maternal diet and adult disease in sheep [11,12,13]. Therefore, this sheep can represent a suitable model for investigating maternal metabolic disorders caused by malnutrition during pregnancy.

Metabolomics is a research method that quantitatively analyzes all small-molecule metabolites with a relative molecular mass of less than 1000 in the organism and explores the relationship between metabolites and physiological and pathological changes [14]. Thus, metabolomics can comprehensively reveal the overall changes in metabolic disorders caused by malnutrition. In the present study, we hypothesized that continuous detection of blood metabolites can reveal the entire metabolic changes of metabolic disorders induced by FR. In addition, we also investigated the pathogenesis of metabolic disorders, which will help to alleviate the metabolic disorders caused by malnutrition.

## 2. Results

### 2.1. LC/MS Compound Identification and Quantification

A total of 578 valid peaks were obtained that were unique and non-overlapping in the blood samples. After rigorous quality control and identification, 252 compounds were relatively quantified. Multivariate analysis was performed to explore the temporal changes of the blood metabolic profile in ewes during late gestation and the effects of FR on it.

### 2.2. Principal Component Analysis (PCA) and Partial Least Squares-Discriminate Analysis (PLS-DA)

**Figure 1:**
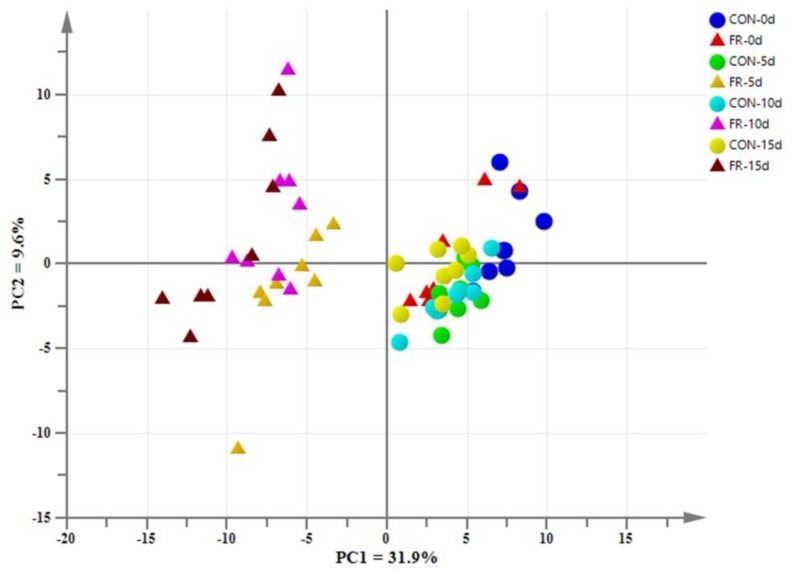
Principal component analysis (PCA) scores plot of blood metabolites based on Liquid Chromatography-Mass Spectrometry (LC/MS) from the control (CON) and feed restriction (FR) groups. CON group: Circle; FR group: Triangle. Blood samples collected before (0 d) and after feed restriction (5 d, 10 d, and 15 d) in CON and FR group.

**Figure 2:**
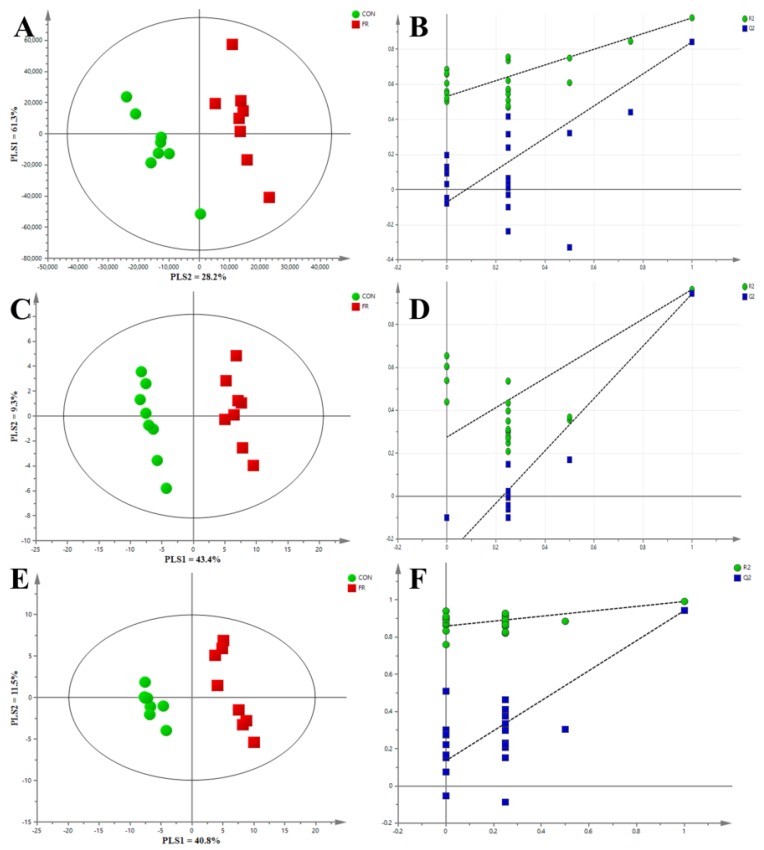
Partial least squares-discriminate analysis (PLS-DA) scores plot of blood metabolites based on LC-MS from the CON and FR groups at day 5 (**A**), day 10 (**C**), and day 15 (**E**). (**B**,**D**,**F**) Validation plot of PLS-DA at day 5, day 10, and day 15, respectively.

The PCA score plot revealed that the first and second principal components (PCs) explained 31.9% and 9.6% of the variation, respectively (Figure 1). There was no clear distinction among the groups of FR-0 d, CON-0 d, CON-5 d, CON-10 d, and CON-15 d. As expected, the PCA revealed clear separations in blood metabolites between before (day 0) and after the treatment (day 5, day 10, and day 15) in FR group (*p* = 0.001). In addition, there was no clear distinction between the FR-5 d and FR-10 d (*p* = 0.11), but there were clear separations between the FR-10 d and FR-15 d (*p* = 0.032), and significant differences between the FR-5 d and FR-15 d (*p* = 0.012) in blood metabolites. Compared with the control (CON) group, the sample distribution was more dispersed and the distance among the samples was farther in the FR-10 d and FR-15 d groups. For further analysis, PLS-DA was carried out to explore the differences between the CON group and FR group at each time point (day 5, day 10, and day 15). As shown in Figure 2, the PLS-DA showed that the blood metabolites of the CON group were clearly distinguished from those of the FR group on day 5 (Figure 2A), day 10 (Figure 2C), and day 15 (Figure 2E). The corresponding values of R2X, R2Y, and Q2 of PLS-DA model are listed in Table S1.

### 2.3. Differences in Blood Metabolites between the CON and FR Groups at Day 5, Day 10, and Day 15

In total, there were 120 differential metabolites at day 5, 129 differential metabolites at day 10, and 114 differential metabolites at day 15 between CON and FR group according to the threshold (FDR < 0.05, FC > 1.5 or < 0.67, and VIP > 1). As shown in Table S2, most fatty acids were upregulated in blood samples in FR group compared with CON group. All triglycerides in detected differential metabolites were up-regulated in FR group compared with CON group except that MG(0:0/24:1(15Z)/0:0) was down-regulated. In addition, all fatty acylcarnitines in detected differential metabolites were up-regulated in FR group compared with CON group, while L-carnitine was down-regulated. Most of the phosphatides were significantly down-regulated in FR group compared with CON group.

**Figure 3:**
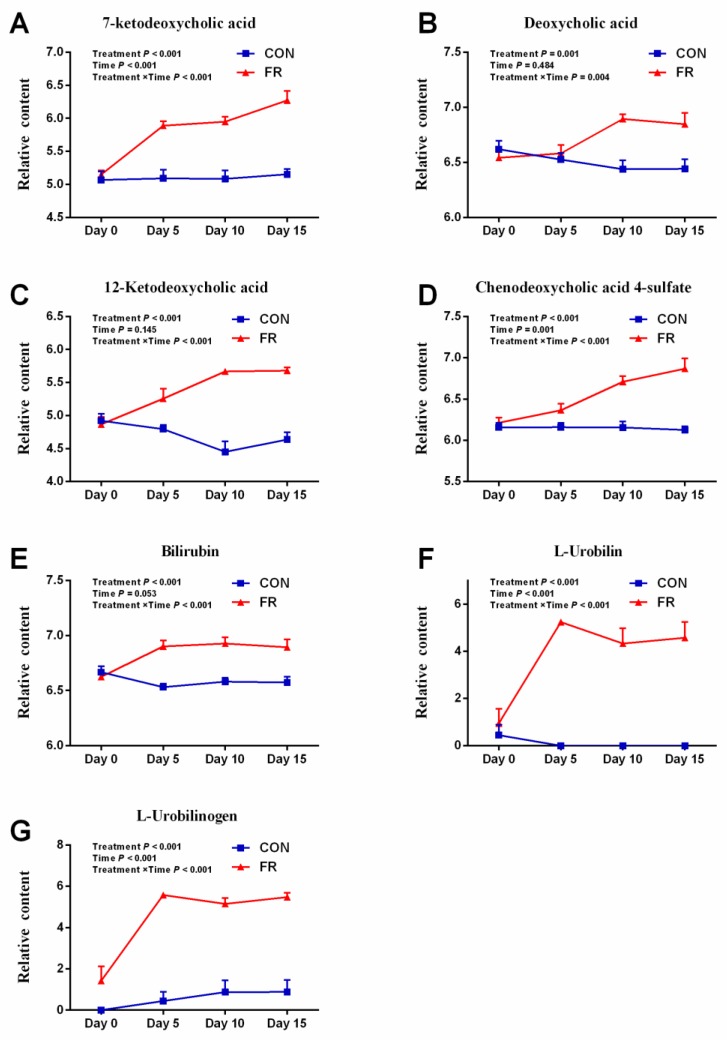
FR increased the blood concentration of bile constituents (bilirubin, bile acids, and cholic acid).

For metabolic intermediates in the glucose metabolism pathway, the level of glucose-6-phosphate, citrate, and α-ketoglutarate were down-regulated in FR group compared with CON group. In details, as treatment time increases, the level of citrate and α-ketoglutarate decreased, while the largest decline in glucose-6-phosphate level occurred on the fifth day of treatment (log_2_ (FC) = −3.30), after which the decline began to decrease (log_2_ (FC) = −2.57 at day 10), and there was no significant difference in the level of glucose-6-phosphate on the 15th day of treatment between the CON and FR group in spite of the value of log_2_ (FC) was −2.84. Interestingly, malate, which could participate in gluconeogenesis process and produce glucose-6-phosphate, was up-regulated with the extension of treatment time. Both ketogenic compounds (leucic acid and lysine) were increased in FR group compared with CON group, while tryptophan, which is also a ketogenic compound, was decreased. However, all of the glycogenic amino acid in detected differential metabolites were down-regulated except for histidine in the FR group compared with the CON group. In addition, FR significantly increased the level of 7-ketodeoxycholic acid, deoxycholic acid, 12-ketodeoxycholic acid, chenodeoxycholic acid 4-sulfate, bilirubin, L-urobilin, and L-urobilinogen (Figure 3).

**Figure 4:**
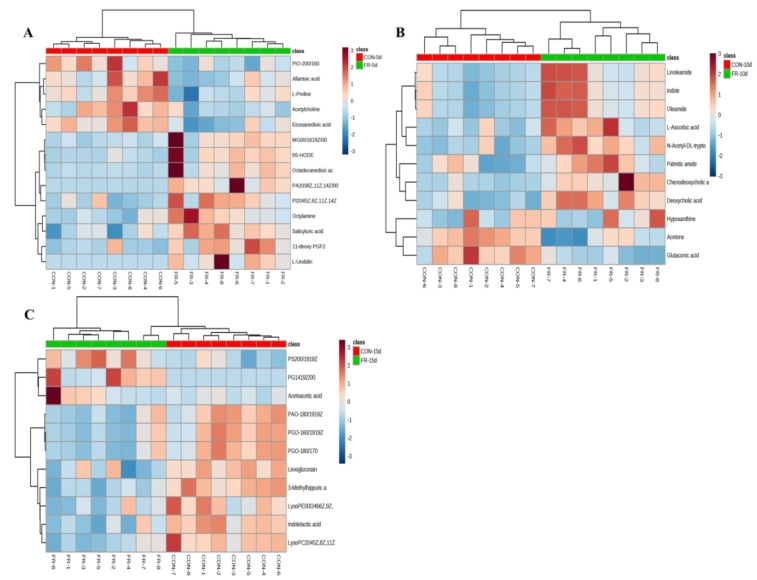
Hierarchical clustering analysis for unique differential metabolites identified in the comparison between CON and FR groups at 5 d (**A**), 10 d (**B**), and 15 d (**C**).

For further understanding the changes of blood metabolites in response to FR, hierarchical clustering analysis (HCA) for the unique differential metabolites identified in the comparison between CON and FR group at 5 d, 10 d, and 15 d was performed (Figure 4). The HCA results indicated that there were five decreased metabolites (acetylcholine, allantoic acid, eicosanedioic acid, l-proline, and PI(O-20:0/16:0)) and nine increased metabolites (11-deoxy PGF2, 9(S)-HODE, L-urobilin, MG(0:0/16:1(9Z)/0:0), octadecanedioic acid, octylamine, PA(20:3(8Z,11Z,14Z)/0:0), PI(20:4(5Z,8Z,11Z,14Z)/0:0), and salicyluric acid) in the FR group compared with the CON group at 5 d. There were two decreased metabolites (acetone and glutaconic acid) and nine increased metabolites (chenodeoxycholic acid 4-sulfate, deoxycholic acid, hypoxanthine, indole, L-ascorbic acid, linoleamide, N-acetyl-DL-tryptophan, oleamide, and palmitic amide) in the FR group compared with the CON group at 10 d. There were eight decreased metabolites (3-methylhippuric acid, indolelactic acid, levoglucosan, LysoPC(20:4(5Z,8Z,11Z,14Z)), LysoPE(0:0/24:6(6Z,9Z,12Z,15Z,18Z,21Z)), PA(O-18:0/19:1(9Z)), PG(O-16:0/19:1(9Z)), and PG(O-18:0/17:0))) and three increased metabolites (acetoacetic acid, PG(14:1(9Z)/0:0), and PS(20:0/19:1(9Z))) in the FR group compared with the CON group at 15 d. These compounds in particular, for each time of sampling, may be important for understanding the process of metabolic changes in response to FR.

### 2.4. Metabolic Pathways of Differential Metabolites

**Figure 5:**
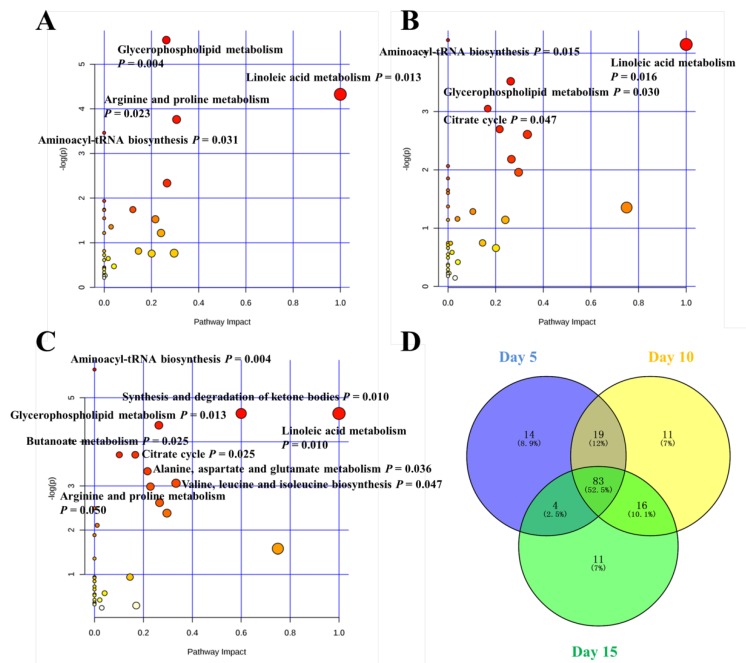
Metabolome view map of the significantly different metabolites identified in the control (CON) and feed restriction (FR) group at 5 d (**A**), 10 d (**B**), and 15 d (**C**). Pathway analysis showed different metabolic pathways were significantly enriched as the feed restriction time increased and mainly related to amino acid metabolism and fatty acid metabolism (A, B, and C). The larger size indicates higher pathway enrichment, and the darker color indicates higher pathway impact values (A, B, and C). Common and unique differential metabolites identified in the comparison between control (CON) and feed restriction (FR) group at 5 d, 10 d, and 15 d (**D**).

**Figure 6:**
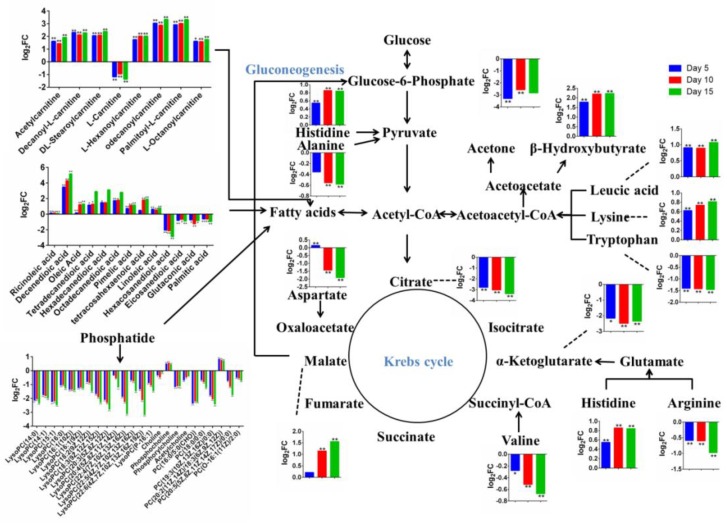
Overview of metabolic alteration induced by severe FR.

In order to comprehensively understand the dynamic adaptation of ewe body metabolism to severe FR, pathway enrichment analysis was performed based on differential metabolites at 5, 10, and 15 d of the treatment (Figure 5). Glycerophospholipid metabolism, linoleic acid metabolism, arginine and proline metabolism, and aminoacyl-tRNA biosynthesis were enriched at day 5. Aminoacyl-tRNA biosynthesis, aminoacyl-tRNA biosynthesis, glycerophospholipid metabolism, and citrate cycle were enriched at day 10. Aminoacyl-tRNA biosynthesis, synthesis and degradation of ketone bodies, glycerophospholipid metabolism, butanoate metabolism, linoleic acid metabolism, citrate cycle, alanine, aspartate and glutamate metabolism, valine, leucine and isoleucine biosynthesis, and arginine and proline metabolism were enriched at day 15. There were 83 common differential metabolites identified in the comparison between CON and FR groups at day 5, day 10, and day 15, and there were 14, 11, and 11 unique differential metabolites between CON and FR group at day 5, day 10, and day 15, respectively (Figure 5D). The overview of metabolic alteration induced by severe FR was performed (Figure 6).

## 3. Discussion

Previous studies in ruminants showed that increased energy demand due to perinatal fetal growth and a sharp increase in lactation results in negative energy balance when energy intake is insufficient [10,15]. The body undergoes adaptive metabolism to maintain the balance of blood glucose, which is important for organs to function normally. The body storage (fatty acids and amino acids) was mobilized to provide energy and participate in gluconeogenesis. However, acetyl-CoA produced by fatty acids β-oxidation produces a large amount of ketone bodies, which can be cytotoxic at high concentrations [3]. In addition, excess lipids are stored in the liver in the form of triglycerides causing fatty liver and impairing liver metabolic functions [3]. In the present study, metabolomics based on LC/MS was performed to monitor the dynamic effects of severe FR on blood metabolites.

### 3.1. PCA and PLS-DA

PCA results showed that the blood samples gather into a cluster from the CON and FR groups before the treatment (CON-0 d vs. FR-0 d), indicating that there was no significant difference between the two groups before the study. For the CON group, there was no clear metric separation of blood samples among 5 d, 10 d, and 15 d. For the FR group, the PCA showed a clear metric separation of blood samples between before (FR-0 d) and after (FR-5 d, FR-10 d, and FR-15 d) the treatment. These results indicated that the treatment of FR has a noticeable impact on the metabolism of the ewes’ body compared with the pregnancy period with the normal energy supply. There was no clear distinction between the FR-5 d and FR-10 d, but there were clear separations between the FR-10 d and FR-15 d, and significant differences between the FR-5 d and FR-15 d in blood metabolites, indicating that sharp changes in blood metabolites have been established between the 5th and 10th days of FR, and it deepened slightly as the treatment persisted. The Venn diagram, which was drawn from the differential metabolites of the two groups at three time points, also illustrates these results. When comparing both groups, there were 120, 129, and 114 differential metabolites at day 5, day 10, and day 114 between the two groups, respectively. Among all these metabolites, there were 83 common differential metabolites in the three time points, which represent 52.5% of the total differential metabolites.

### 3.2. Fatty Acid Metabolism

Compared with other macromolecular substances, lipids stored in adipose tissue have advantages, including low water content and high energy density, as physiological fuels [16]. In the present study, most of the fatty acids and triglycerides were increased in blood samples induced by severe FR. A previous study indicated that concentrations of metabolites (such as fatty acids and triglycerides) in the blood circulation can reflect the mobilization of body lipids in negative energy balance [16]. Thus, the results of the present study suggested that the body lipids mobilization existed on the fifth day of severe FR treatment, and deeper mobilization of lipids is noticed with the extension of the treatment time. Subsequently, the mobilized fatty acids should be transferred across the inner mitochondrial membrane for subsequent β-oxidation by carnitine, especially long-chain fatty acids. In the present study, all the fatty acylcarnitines were increased in blood samples in the FR group, while the level of L-carnitine was decreased. However, our previous study of liver metabolomics profiling showed the increased L-carnitine level in liver [5]. In line with our study, Schooneman et al. (2013) demonstrated that increased fatty acids absorbed in the liver can lead to accumulation of fatty acylcarnitines [17], and it is a detoxification process in the liver to release them into the blood circulation [18]. In addition, previous study showed that FR could decrease endogenous synthesis of carnitine [19], while increasing hepatic carnitine concentration [20], and the reason should be attributed to changes in hormones induced by FR [19]. A study in rat liver has also shown that the net uptake rate of liver in the fasted state increased by 62.5% compared to those in fed state [21]. Thus, the results in the present study suggested that more L-carnitine was absorbed into the liver for playing a key role in the transport of long-chain fatty acids into mitochondria. Interestingly, in the present study, most choline were decreased in blood samples, while they were increased in the liver [20]. Choline exist in the form of the phospholipids phosphatidylcholine (PC) and lysophosphatidylcholine, which could be assembled into very low-density lipoprotein (VLDL) with triglyceride and then transported out of the liver [22], which could alleviate fat deposition in the liver. Thus, although more choline enters the liver, the triglyceride content in the liver did not decrease but rather, increased, and fatty liver was aggravated. Given that ruminants have an inherently low hepatic capacity to synthesize VLDL, rumen-protected choline supplement could increase the transport of fatty acids out of the liver, which can be explained by the provision of methyl groups and improving hepatic intake of carnitine induced by choline [22]. These results suggested that lipid mobilization was induced by FR and body metabolism changed in response to the challenge, including increased transport of choline and carnitine into the liver, but it was not enough to alleviate fat deposition in the liver.

### 3.3. Amino Acid Metabolism

Amino acids derived from muscle were important gluconeogenesis substrates in starvation [23]. Both ketogenic compounds (leucic acid and lysine) were increased in the FR group compared with the CON group, while tryptophan, which is also a ketogenic compound, was decreased. All of the glycogenic amino acids were down-regulated except for histidine in the FR group compared with the CON group. The three urea-cycle amino acids, arginine, citrulline, and ornithine were also decreased. A previous study indicated that starving animals showed different changes in circulating amino acids [16]. The decreased glycogenic amino acid levels indicated that they were used as substrates to enter the tricarboxylic acid cycle to provide energy or participate in the gluconeogenesis process to produce glucose [16]. Given that fatty acids mobilized from fat tissue and the ketogenic amino acids could be converted to BHBA, the increased ketogenic amino acids levels in the present study probably indicated that excess BHBA feedback inhibit the process. In line with these findings in the present study, Bergman et al. also demonstrated the decreased level of the three urea cycle amino acids, which were attributed to decrease in ammonia absorption induced by low feed intake [24].

### 3.4. Bile Constituents

In the present study, the blood concentration of bile constituents (bilirubin, bile acids, and cholic acid) were increased in the FR group (Figure 3). Previous studies reported decreased bile flow in ruminants with fatty liver [3]. In addition, given that the high concentrations of bile are toxic and increase the production of free radicals in the liver, which can cause inflammation and tissue damage [3], excessive bilirubin content in the blood has been reported to be neurotoxic [25], but the relationship between bilirubin and an irreversible encephalopathy hypoglycemic brain injury in ruminants in negative energy balance [3,26] was not clear.

### 3.5. Metabolic Pathway

This study also explored the dynamic response of metabolic pathways to severe FR. It has been established that animal physiological fuels are dominated from carbohydrate shifting to lipid or protein during starvation [16]. In the present study, four metabolic pathways (glycerophospholipid metabolism, linoleic acid metabolism, arginine and proline metabolism, and aminoacyl-tRNA biosynthesis) at day 5 were different, and the pathway with the greatest impact value was linoleic acid metabolism (impact value = 1). At day 10, the differential pathway analysis had a newly added citrate cycle (TCA) pathway. At day 15, more pathways related to amino acid metabolism were different in spite of the fact that metabolic pathways related to fatty acid have higher impact values. Given that the TCA cycle is the final common oxidative pathway for carbohydrates, fats, and amino acids, the results in the present study suggested that the FR mainly affects pathways related to fatty acid in the early stage, followed by TCA cycle pathway, and pathways related to amino acid and, ultimately, metabolic pathways related to the three substances.

Previous study indicated that the physiological switch from lipid-dominated catabolism to protein-dominated catabolism is thought to occur only when an animal’s lipid levels reach some critical threshold [16]. The results of this study showed disturbed fatty acid oxidation, including over-mobilized adipose tissue, increased blood BHBA level, and insufficient choline. In addition, the acetyl-CoA produced by the oxidation of fatty acids did not effectively enter the TCA cycle to produce energy, which, in turn, produced ketone bodies, evidenced by decreased citrate content and increased BHBA content, which probably attributed to lack of sufficient oxaloacetate to combine with acetyl-CoA. In this case, amino acids may be more readily utilized to enter the TCA cycle and participate in the gluconeogenesis pathway to produce glucose. The up-regulated malate illustrates this speculation, which can be converted from amino acids entering the TCA cycle and converted to glucose-6-phosphate. The reduction trend of glucose-6-phosphate has been alleviated in the present study (Figure 6). Therefore, the key to solving the metabolic disorder caused by insufficient energy was probably to make acetyl-CoA enter the TCA cycle effectively, such as providing enough oxaloacetate or amino acid that can be converted to oxaloacetate (aspartic acid), which could reduce ketone bodies content generated by acetyl-CoA and decrease the oxidation of amino acid.

## 4. Materials and Methods

### 4.1. Animals and Experimental Design

The experimental design and procedures of this study were approved by the Animal Care and Use Committee of Nanjing Agricultural University following the requirements of the Regulations for the Administration of Affairs Concerning Experimental Animals (The State Science and Technology Commission of P. R. China, 1988. No. SYXK(Su)2015-0656). The current study is a continuation of previous research, where the effect of feed restriction on lipid metabolism disorder in livers of ewes was investigated [4]. The experiment design and treatments are described in detail [10]. Briefly, 20 pregnant Hu sheep (body weight 60.6 ± 4.9 kg) carrying multiple fetuses (an average of 2.7 fetuses per ewes, and no significant difference between the two groups) with a gestation period of 108 days and the same parity (2 to 3 parity) were selected. After 7 days of adaption period, they were randomly divided into two groups, the control group (CON) were offered 100% of their National Research Council (NRC)-recommended nutritional requirements (1.56 kg total mixed ration, dry matter basis) and the severe feed restriction group (FR) were restricted to 30% of the control group (0.47 kg total mixed ration). During the treatment period, the Hu sheep were housed in individual pens with free access to water. Ingredients and nutritional compositions of the total mixed ration are presented in Table S3.

### 4.2. Samples Collection

Jugular vein blood was collected using blood collection tubes, containing 40 KIU Na-heparin/mL blood before morning feeding on day 0 before the treatment and on days 5, 10, and 15 of the treatment period. Blood samples were centrifuged at 3000× *g* for 10 min and the supernatant was transferred to a cryotube and stored in liquid nitrogen.

### 4.3. Liquid Chromatography-Mass Spectrometry (LC/MS) Analysis

Blood samples from 8 ewes, which were randomly selected from the two groups, were used for metabolomics analysis. The method of sample preparation for LC/MS detection are described in detail [5]. Briefly, 100 μL of blood samples, 0.3 mL methanol (Merck, Dannstadt, Germany), and 10 μL L-2-chlorophenylalanine (interior label) were added to a 1.5 mL Eppendorf tube. This mixture was vortexed for 30 s (Votex-5, Kylin-Bell Lab Instruments Co., LTD., Haimen, China), then centrifuged at 13,800 *g* for 10 min at 4 °C, and 200 μL of the supernatant was transferred to the injection vial. A total of 4 μL blood samples at 4 °C were injected to the LC–MS system (Thermo, Ultimate 3000LC, Orbitrap Elite) with an Agilent C18 column (Hypergod C18, 100 mm × 4.6 mm × 3 μm) and the column temperature was maintained at 40 °C. The mobile phase consisted of water with 0.1% (*v*/*v*) formic acid (A) and acetonitrile with 0.1% (*v*/*v*) formic acid (B) and the flow rate was 0.3 mL/min. The blood samples were eluted with a gradient of 5% B for 0−2 min, 5%−95% B for 2−12 min, 95% B for 12−15 min, and 95%−5% B for 15−17 min. The spray voltage was 3.0 kV. The heater temperature and the capillary temperature were 300 °C and 350 °C, respectively. The sheath gas flow rate, aux gas flow rate, and sweep gas flow rate were 45 arb, 15 arb, and 1 arb, respectively. The Kyoto Encyclopedia of Genes and Genomes Database (http://www.genome.jp/kegg) and the online Human Metabolome Database (http://www.hmdb.ca) were utilized to align the molecular mass data to identify metabolites. The metabolite name was reported if the difference between theoretical mass and observed mass was less than 10 ppm. Then, the method of isotopic distribution measurement was used to further validate these matched metabolites. Commercial reference standards were utilized to validate and confirm the blood metabolites with high confidence by comparison of their MS/MS spectra and retention time. The raw data was preprocessed by SIEVE software (ThermoFisher Scientific, Waltham, MA, USA) for LC/MS data, and normalized and post-edited in EXCEL 2010 software. The final result was changed to 2 D data matrix, including variance (Rt/mz), observed quantity, and peak intensities.

### 4.4. Data Analysis

The final data were imported into SIMCA-P software (Version 13, Umetrics AB, Sweden) for multivariate statistical analysis. PCA and PLS-DA were carried out to explore the differences of the metabolites between the two groups. PERMANOVA analysis was performed in order to calculate the distance significance of the PCA plot using Vegan R-Package (Adonis). The false discovery rate (FDR), fold change (FC, FR/CON), and the values of variable importance in the projection (VIP) in the PLS-DA model were used as indicators to evaluate differential metabolites in both groups (FDR < 0.05, FC > 1.5 or < 0.67, and VIP > 1). Differential metabolite data were used for pathway enrichment analysis on the MetaboAnalyst 3.0 (http://www.metaboanalyst.ca). *P*-values < 0.05 were considered statistically significant.

## 5. Conclusions

In the present study, severe FR resulted in a noticeable disorder in fatty acid oxidation metabolism due to lipid mobilization in the absence of energy. Some toxic metabolites related to fatty liver, brain injury, and tissue damage were produced due to these metabolic disorders. We recommend directing some research towards finding some innovative ways to get acetyl-CoA into the TCA cycle.

## Supplementary Materials

The following are available online at https://www.mdpi.com/2218-1989/9/6/112/s1. Table S1. The values of R2X, R2Y, and Q2 in PLSDA model. Table S2. Identification of significantly different metabolites in blood between ewes from CON and FR group. Table S3 Ingredients and nutrient composition of the experimental diets for pregnant sheep (Ovis aries) in this study.
