# Supplementary material for: Dynamic Changes of Plasma Metabolome in Response to Severe Feed Restriction in Pregnant Ewes
Source: Metabolites. 2019 Jun 10;9(6):112. doi: 10.3390/metabo9060112 (PMC6630903; doi:10.3390/metabo9060112)
Supplement: Supplementary file 1 [file metabolites-09-00112-s001.pdf]

Supplementary Materials:

Table S1 The values of R2X, R2Y, and Q2 in PLSDA model.

	model	R2X	R2Y	Q2
Day 5	PLSDA	0.917	0.978	0.842
Day 10	PLSDA	0.434	0.964	0.944
Day 15	PLSDA	0.62	0.992	0.943

Table S2. Identification of significantly different metabolites in blood between ewes from CON and FR group.

ID	MZ	Time	Day 5					Day 10					Day 15				
			VIP	Fold Change	log_2(FC)	p value	FDR	VIP	Fold Change	log_2(FC)	p value	FDR	VIP	Fold Change	log_2(FC)	p value	FDR
**Fatty acid metabolism**																	
Decenedioic acid	221.08	4.26	1.34	11.14	3.48	<0.001	<0.001	1.24	19.46	4.28	<0.001	<0.001	1.15	36.08	5.17	<0.001	<0.001
12-Ketodeoxycholic acid	389.27	6.37	—	—	—	—	—	1.42	11.80	3.56	<0.001	<0.001	1.39	9.08	3.18	<0.001	<0.001
15d-PGA1	317.21	7.38	1.14	4.35	2.12	0.008	0.020	1.04	5.76	2.53	0.004	0.009	—	—	—	—	—
7-ketodeoxycholic acid	405.26	5.06	1.23	4.72	2.24	<0.001	0.002	1.14	5.51	2.46	0.002	0.005	—	—	—	—	—
b-hydroxybutyric acid	105.03	3.69	1.50	3.45	1.78	<0.001	<0.001	1.41	4.61	2.20	<0.001	<0.001	1.43	4.70	2.23	<0.001	<0.001
Decanoyl-L-carnitine	316.25	4.93	1.00	4.98	2.31	<0.001	0.001	1.37	4.35	2.12	<0.001	<0.001	1.16	4.87	2.28	<0.001	0.002
DL-Stearoylcarnitine	428.37	8.40	1.44	4.18	2.06	<0.001	<0.001	1.46	4.25	2.09	<0.001	<0.001	1.44	5.24	2.39	<0.001	<0.001
L-Hexanoylcarnitine	260.19	3.91	1.14	3.37	1.75	<0.001	0.001	1.42	4.06	2.02	<0.001	<0.001	1.54	4.05	2.02	<0.001	<0.001
10(S)-HpODE	313.24	7.42	1.28	2.43	1.28	0.003	0.008	1.38	3.27	1.71	<0.001	<0.001	—	—	—	—	—
L-Octanoylcarnitine	288.22	4.44	—	—	—	—	—	1.33	3.02	1.59	<0.001	<0.001	1.19	3.38	1.76	<0.001	0.002
Hexadecanedioic acid	285.21	6.68	1.15	2.80	1.48	0.015	0.033	1.23	2.80	1.49	0.010	0.020	—	—	—	—	—
Acetylcarnitine	202.11	3.50	1.50	3.07	1.62	<0.001	<0.001	1.20	2.71	1.44	<0.001	0.002	1.06	3.79	1.92	0.002	0.005
13(S)-HpOTrE	309.21	6.95	1.29	2.18	1.12	0.005	0.012	1.21	2.39	1.26	0.010	0.020	—	—	—	—	—
2-Hydroxymyristic Acid	243.20	8.29	1.30	2.25	1.17	0.002	0.005	1.23	2.23	1.15	0.001	0.003	—	—	—	—	—
Linoleamide	280.26	9.22	—	—	—	—	—	1.02	2.00	1.00	0.002	0.005	—	—	—	—	—
D-Glycerol 1-phosphate	171.03	1.39	1.55	1.85	0.89	<0.001	<0.001	1.48	1.93	0.95	<0.001	<0.001	1.45	1.75	0.81	<0.001	<0.001

3-Hydroxyisovaleric acid	117.06	1.92	1.20	1.34	0.42	0.003	0.008	1.41	1.79	0.84	<0.001	<0.001	1.42	1.95	0.96	<0.001	<0.001
Linoleic acid	281.25	7.85	1.30	1.56	0.64	<0.001	0.001	1.03	1.45	0.54	0.005	0.010	1.06	1.73	0.79	<0.001	<0.001
Levonorgestrel	311.20	8.48	1.23	1.04	0.06	0.002	0.005	1.28	1.05	0.07	<0.001	<0.001	1.10	1.03	0.05	0.007	0.014
2-Tocotrienol	423.33	11.71	1.21	0.95	-0.07	<0.001	0.001	1.07	0.97	-0.05	0.005	0.010	—	—	—	—	—
2-ketoisovaleric acid	117.06	3.40	—	—	—	—	—	1.28	0.96	-0.06	<0.001	<0.001	1.00	0.93	-0.10	0.001	0.003
5-Methoxycinnamic acid	177.09	5.68	—	—	—	—	—	1.01	0.90	-0.15	0.005	0.010	1.20	0.84	-0.25	<0.001	0.001
DHAP(18:0)	435.25	8.68	—	—	—	—	—	1.10	0.82	-0.29	0.005	0.010	1.19	0.73	-0.46	<0.001	0.002
Choline	104.11	10.84	1.09	0.79	-0.34	0.010	0.024	1.31	0.71	-0.49	<0.001	<0.001	1.37	0.91	-0.14	<0.001	<0.001
Acetone	57.55	1.22	—	—	—	—	—	1.11	0.71	-0.49	<0.001	0.002	—	—	—	—	—
DG(18:4(6Z,9Z,12Z,15Z)/15:0/0:0)	288.64	8.72	1.28	0.69	-0.54	<0.001	0.001	1.22	0.65	-0.62	0.001	0.003	1.21	0.61	-0.72	0.001	0.003
L-Carnitine	162.11	0.87	1.52	0.45	-1.17	<0.001	<0.001	1.30	0.52	-0.94	<0.001	0.001	1.37	0.39	-1.36	<0.001	0.001
Gentisic acid	153.02	3.79	1.28	0.43	-1.22	<0.001	0.002	1.23	0.47	-1.08	0.002	0.005	1.35	0.28	-1.85	<0.001	<0.001
Glucoheptonic acid	227.08	3.38	1.57	0.42	-1.24	<0.001	<0.001	1.46	0.45	-1.16	<0.001	<0.001	1.46	0.44	-1.20	<0.001	<0.001
Glutaconic acid	227.00	0.91	—	—	—	—	—	1.27	0.43	-1.22	0.001	0.003	—	—	—	—	—
DG(P-14:0/18:1(9Z))	552.33	5.64	1.37	0.11	-3.17	<0.001	<0.001	1.13	0.19	-2.36	<0.001	0.001	—	—	—	—	—
11-deoxy PGF2	337.24	6.69	1.11	2.55	1.35	0.007	0.017	—	—	—	—	—	—	—	—	—	—
9(S)-HODE	295.23	7.34	1.10	2.28	1.19	0.007	0.017	—	—	—	—	—	—	—	—	—	—
Acetoacetic acid	103.04	4.13	—	—	—	—	—	—	—	—	—	—	1.07	0.51	-0.97	0.021	0.042
Acetylcholine	146.12	0.88	1.13	0.61	-0.70	0.007	0.017	—	—	—	—	—	—	—	—	—	—
CPA(18:2(9Z,12Z)/0:0)	417.28	4.76	1.25	0.29	-1.81	<0.001	<0.001	—	—	—	—	—	1.12	0.18	-2.51	0.002	0.005
Eicosanedioic acid	341.27	8.46	1.20	0.58	-0.79	0.003	0.008	—	—	—	—	—	—	—	—	—	—
Hexacosanedioic acid	425.36	12.01	1.58	0.24	-2.04	<0.001	<0.001	—	—	—	—	—	1.49	0.14	-2.87	<0.001	<0.001
LysoPC(22:5(4Z,7Z,10	570.35	7.80	1.14	0.62	-0.69	0.007	0.017	1.01	0.59	-0.77	0.003	0.006	1.33	0.36	-1.49	<0.001	<0.001

Z,13Z,16Z))																	
LysoPC(18:2(9Z,12Z))	520.34	7.59	1.50	0.57	-0.81	<0.001	<0.001	1.47	0.54	-0.88	<0.001	<0.001	1.52	0.35	-1.50	<0.001	<0.001
LysoPC(P-16:1)	480.34	9.04	1.39	0.54	-0.89	<0.001	<0.001	1.37	0.48	-1.06	<0.001	<0.001	1.46	0.36	-1.47	<0.001	<0.001
LysoPC(16:0)	496.34	8.18	1.54	0.48	-1.07	<0.001	<0.001	1.42	0.49	-1.03	<0.001	<0.001	1.50	0.41	-1.28	<0.001	<0.001
LysoPC(16:1(9Z))	538.31	8.02	1.43	0.42	-1.25	<0.001	<0.001	1.33	0.44	-1.19	<0.001	<0.001	1.32	0.41	-1.27	<0.001	<0.001
LysoPC(22:6(4Z,7Z,10 Z,13Z,16Z,19Z))	568.34	7.61	1.25	0.39	-1.35	<0.001	0.002	1.32	0.23	-2.13	<0.001	<0.001	1.44	0.11	-3.20	<0.001	0.003
LysoPC(16:1(10Z))	494.32	7.43	1.49	0.38	-1.38	<0.001	<0.001	1.33	0.39	-1.37	<0.001	<0.001	1.35	0.37	-1.43	<0.001	<0.001
LysoPC(18:3(9Z,12Z,15 Z))	518.32	7.17	1.60	0.31	-1.68	<0.001	<0.001	1.45	0.26	-1.92	<0.001	<0.001	1.50	0.20	-2.32	<0.001	<0.001
LysoPC(14:1)	468.31	7.08	1.52	0.29	-1.77	<0.001	<0.001	1.37	0.28	-1.83	<0.001	<0.001	1.35	0.25	-2.01	<0.001	<0.001
LysoPC(22:4(7Z,10Z,13 Z,16Z))	544.34	7.70	1.48	0.27	-1.89	<0.001	<0.001	1.36	0.21	-2.26	<0.001	<0.001	1.52	0.11	-3.12	<0.001	<0.001
LysoPC(20:3(5Z,8Z,12 Z))	546.35	8.23	1.49	0.23	-2.11	<0.001	<0.001	1.33	0.20	-2.33	<0.001	<0.001	1.37	0.14	-2.81	<0.001	<0.001
LysoPC(14:0)	466.29	7.29	1.51	0.22	-2.16	<0.001	<0.001	1.39	0.24	-2.08	<0.001	<0.001	1.40	0.20	-2.35	<0.001	<0.001
LysoPC(15:1)	482.32	7.71	1.58	0.21	-2.26	<0.001	<0.001	1.39	0.21	-2.22	<0.001	<0.001	1.43	0.18	-2.49	<0.001	<0.001
LysoPC(20:4(5Z,8Z,11 Z,14Z))	544.34	7.70	—	—	—	—	—	—	—	—	—	—	1.27	0.43	-1.20	<0.001	0.002
LysoPE(0:0/18:0)	480.31	8.54	1.47	0.67	-0.57	<0.001	<0.001	1.45	0.65	-0.61	<0.001	<0.001	1.47	0.53	-0.92	<0.001	<0.001
LysoPE(0:0/20:3(11Z,14 Z,17Z))	502.29	7.27	1.15	1.24	0.32	0.010	0.024	1.44	0.32	-1.62	<0.001	<0.001	1.48	0.26	-1.97	<0.001	<0.001
LysoPE(0:0/20:0)	508.34	17.15	1.46	0.64	-0.63	<0.001	<0.001	1.38	0.62	-0.68	<0.001	<0.001	1.50	0.46	-1.12	<0.001	<0.001
LysoPE(0:0/20:5(5Z,8Z, 11Z,14Z,17Z))	498.26	6.85	1.03	0.54	-0.90	0.010	0.024	1.11	0.53	-0.92	0.010	0.020	1.05	0.43	-1.21	0.007	0.014
LysoPE(0:0/18:2(9Z,13	476.28	7.42	1.44	0.46	-1.11	<0.001	<0.001	1.18	0.57	-0.81	0.003	0.007	1.24	0.46	-1.12	<0.001	0.002

Z))																	
LysoPE(18:2(9Z,12Z)/0:0)	478.29	7.42	1.46	0.45	-1.17	<0.001	<0.001	1.15	0.53	-0.92	0.005	0.010	1.26	0.44	-1.19	<0.001	0.002
LysoPE(0:0/20:1(12Z))	508.34	8.02	1.39	0.43	-1.23	<0.001	0.001	1.23	0.44	-1.18	0.001	0.003	1.27	0.42	-1.26	<0.001	0.002
LysoPE(18:3(6Z,9Z,12Z)/0:1)	474.26	6.84	1.46	0.33	-1.62	<0.001	<0.001	1.34	0.36	-1.49	<0.001	0.001	1.26	0.31	-1.68	<0.001	0.002
LysoPE(0:0/16:0)	452.28	7.08	1.45	0.32	-1.64	<0.001	<0.001	1.37	0.32	-1.67	<0.001	<0.001	1.34	0.26	-1.92	<0.001	<0.001
LysoPE(0:0/22:5(4Z,7Z,10Z,13Z,16Z))	526.29	6.91	—	—	—	—	—	1.04	0.59	-0.76	0.010	0.020	1.26	0.43	-1.22	0.002	0.005
LysoPE(0:0/24:6(6Z,9Z,12Z,15Z,18Z,21Z))	552.31	7.61	—	—	—	—	—	—	—	—	—	—	1.02	0.63	-0.68	0.010	0.019
Mevalonic acid	147.07	1.40	1.20	1.98	0.98	<0.001	0.002	1.26	2.74	1.46	<0.001	<0.001	1.13	3.51	1.81	<0.001	<0.001
MG(0:0/20:4(5Z,8Z,11Z,14Z)/0:0)	377.27	6.17	1.39	5.25	2.39	<0.001	<0.001	1.22	5.36	2.42	<0.001	0.002	1.25	3.62	1.86	0.015	0.025
MG(0:0/18:2(9Z,12Z)/0:0)	356.28	5.92	1.21	3.44	1.78	<0.001	<0.001	1.40	3.16	1.66	<0.001	<0.001	1.14	3.74	1.90	<0.001	<0.001
MG(0:0/22:1(13Z)/0:0)	411.18	4.33	1.07	2.77	1.47	0.010	0.024	1.02	2.54	1.34	0.007	0.014	1.04	3.14	1.65	0.005	0.010
MG(0:0/16:1(9Z)/0:0)	327.25	8.32	1.03	2.32	1.21	0.007	0.017	—	—	—	—	—	—	—	—	—	—
MG(0:0/18:3(6Z,9Z,12Z)/0:0)	487.22	11.94	1.36	1.53	0.62	<0.001	0.002	1.17	1.39	0.48	0.002	0.005	1.06	1.39	0.48	0.005	0.010
MG(0:0/24:1(15Z)/0:0)	439.38	12.95	1.56	0.38	-1.41	<0.001	<0.001	1.48	0.32	-1.66	<0.001	<0.001	1.50	0.18	-2.44	<0.001	<0.001
odecanoylcarnitine	344.28	6.00	1.25	8.24	3.04	<0.001	<0.001	1.44	7.37	2.88	<0.001	<0.001	1.03	10.12	3.34	<0.001	<0.001
Oleic Acid	281.25	8.72	1.43	1.13	0.17	<0.001	0.002	1.39	2.33	1.22	<0.001	<0.001	1.36	2.45	1.29	<0.001	<0.001
N-Acetyl-DL-tryptophan	245.10	3.59	—	—	—	—	—	1.21	2.33	1.22	0.001	0.003	—	—	—	—	—
Oleamide	282.28	10.14	—	—	—	—	—	1.06	1.74	0.80	0.003	0.006	—	—	—	—	—

Nonanedioic acid	187.01	2.68	1.49	0.57	-0.82	<0.001	<0.001	1.33	0.64	-0.65	<0.001	<0.001	—	—	—	—	—
Oxaloglutarate	203.02	4.24	1.53	0.12	-3.06	<0.001	<0.001	1.43	0.13	-2.98	<0.001	<0.001	1.43	0.11	-3.15	<0.001	<0.001
Octadecanedioic acid	313.24	7.98	1.08	3.33	1.74	0.001	0.003	—	—	—	—	—	—	—	—	—	—
Octylamine	130.16	3.63	1.09	1.05	0.07	0.007	0.017	—	—	—	—	—	—	—	—	—	—
PA(20:3(8Z,11Z,14Z)/0:0)	459.26	4.66	1.10	54.40	5.77	<0.001	0.003	—	—	—	—	—	—	—	—	—	—
PA(6:0/6:0)	367.16	10.30	1.30	4.09	2.03	0.001	0.003	1.23	5.22	2.39	<0.001	<0.001	—	—	—	—	—
PA(14:0/0:0)	381.19	11.94	1.36	1.66	0.73	<0.001	0.002	1.13	1.49	0.58	0.003	0.007	1.03	1.52	0.61	0.015	0.025
PA(16:0/0:0)	409.23	8.44	1.43	0.68	-0.55	<0.001	<0.001	1.45	0.67	-0.57	<0.001	<0.001	1.46	0.53	-0.91	<0.001	<0.001
PA(12:0/18:4(6Z,9Z,12Z,15Z))	611.49	9.72	1.35	0.51	-0.96	<0.001	0.001	1.36	0.40	-1.34	<0.001	<0.001	1.41	0.25	-2.03	<0.001	<0.001
PA(18:4(6Z,9Z,12Z,15Z)/0:0)	429.21	3.79	1.45	0.15	-2.77	<0.001	<0.001	1.37	0.18	-2.49	<0.001	<0.001	1.41	0.11	-3.22	<0.001	0.003
PA(20:0/0:0)	465.30	16.14	—	—	—	—	—	1.24	1.76	0.81	<0.001	<0.001	1.22	2.19	1.13	<0.001	<0.001
PA(20:4(5Z,8Z,11Z,14Z)e/2:0)	485.27	16.35	—	—	—	—	—	1.19	1.39	0.48	0.002	0.005	1.12	1.42	0.50	0.002	0.005
PA(O-18:0/19:1(9Z))	703.57	17.11	—	—	—	—	—	—	—	—	—	—	1.09	0.81	-0.30	0.007	0.015
Palmitic acid	255.23	8.48	1.48	0.66	-0.60	<0.001	<0.001	1.44	0.65	-0.62	<0.001	<0.001	1.47	0.52	-0.94	<0.001	<0.001
Palmitic amide	255.23	8.48	—	—	—	—	—	1.09	1.52	0.61	0.003	0.006	—	—	—	—	—
Palmitoyl-L-carnitine	400.34	7.66	1.53	7.59	2.92	<0.001	<0.001	1.48	8.08	3.02	<0.001	<0.001	—	—	—	—	—
PC(20:2(11Z,14Z)/18:3(6Z,9Z,12Z))	830.56	10.91	1.17	1.82	0.86	0.005	0.012	1.15	1.72	0.78	0.001	0.003	1.00	1.67	0.74	<0.001	0.002
PC(O-16:1(11Z)/2:0)	522.35	8.72	1.16	0.70	-0.51	0.003	0.008	1.12	0.68	-0.56	0.007	0.014	1.18	0.60	-0.73	0.005	0.011
PC(18:0/0:0)	524.37	10.60	1.44	0.62	-0.68	<0.001	<0.001	1.46	0.56	-0.82	<0.001	<0.001	1.49	0.43	-1.23	<0.001	<0.001
PC(20:5(5Z,8Z,11Z,14Z,17Z)/0:0)	542.32	7.08	—	—	—	—	—	1.21	0.45	-1.16	0.007	0.014	1.36	0.29	-1.78	<0.001	0.002

PC(19:3(10Z,13Z,16Z)/0:0)	530.32	8.61	1.40	0.29	-1.78	<0.001	<0.001	1.31	0.24	-2.06	<0.001	<0.001	1.34	0.18	-2.44	<0.001	<0.001
PC(16:0/5:0(CHO))	592.36	9.18	1.51	0.19	-2.37	<0.001	<0.001	1.39	0.21	-2.23	<0.001	<0.001	1.33	0.21	-2.26	<0.001	0.001
PE(18:1(9E)/18:1(9E))	742.53	7.84						1.09	1.36	0.44	0.005	0.010	1.33	1.50	0.58	<0.001	<0.001
PE(P-16:0/0:0)	464.31	9.02	1.23	0.71	-0.49	<0.001	<0.001	—	—	—	—	—	1.27	0.66	-0.59	<0.001	0.002
PE(P-18:0/0:1)	464.31	9.02	1.25	0.67	-0.57	<0.001	<0.001	1.24	0.67	-0.59	0.001	0.003	1.44	0.50	-1.00	<0.001	<0.001
PG(12:0/0:0)	429.37	15.67	—	—	—	—	—	1.06	0.99	-0.01	0.007	0.014	1.03	216.12	7.76	0.004	0.008
PG(14:1(9Z)/0:0)	453.23	4.16	—	—	—	—	—	—	—	—	—	—	1.03	224.75	7.81	<0.001	0.002
PG(O-16:0/19:1(9Z))	747.56	17.13	—	—	—	—	—	—	—	—	—	—	1.13	0.78	-0.35	0.003	0.007
PG(O-18:0/17:0)	748.57	17.13	—	—	—	—	—	—	—	—	—	—	1.14	0.78	-0.35	0.003	0.007
Phosphocholine	184.07	10.91	1.34	1.43	0.52	<0.001	0.002	1.41	1.49	0.58	<0.001	<0.001	1.33	1.39	0.47	<0.001	<0.001
Phosphorylcholine	170.06	3.31	1.55	0.44	-1.19	<0.001	<0.001	1.42	0.46	-1.12	<0.001	<0.001	1.43	0.46	-1.13	<0.001	<0.001
PI(20:4(5Z,8Z,11Z,14Z)/0:0)	619.29	7.69	1.08	1.35	0.43	0.007	0.017	—	—	—	—	—	—	—	—	—	—
PI(O-20:0/16:0)	855.74	16.36	1.21	1.71	0.78	0.001	0.003	—	—	—	—	—	—	—	—	—	—
Pimelic acid	159.07	3.67	1.07	1.66	0.73	0.005	0.012	1.33	2.17	1.12	<0.001	0.001	1.10	2.28	1.19	<0.001	0.001
PS(16:0/0:1)	498.34	8.47	1.52	0.55	-0.87	<0.001	<0.001	1.47	0.54	-0.90	<0.001	<0.001	1.50	0.41	-1.30	<0.001	<0.001
PS(19:0/0:0)	538.31	7.39	1.43	0.42	-1.25	<0.001	<0.001	1.33	0.44	-1.19	<0.001	<0.001	1.32	0.41	-1.28	<0.001	<0.001
PS(20:0/19:1(9Z))	830.59	17.12	—	—	—	—	—	—	—	—	—	—	1.09	1.16	0.21	0.005	0.010
PS(8:0/8:0)	510.25	4.29	1.17	3.47	1.80	<0.001	0.001	1.05	6.48	2.70	<0.001	<0.001	1.02	7.52	2.91	0.001	0.003
Ricinoleic acid	315.25	7.07	1.32	1.11	0.15	<0.001	<0.001	1.15	1.09	0.13	<0.001	0.001	1.08	1.08	0.11	<0.001	0.001
Salicyluric acid	194.08	6.50	1.23	1.04	0.06	<0.001	0.002	—	—	—	—	—	—	—	—	—	—
tetracosahexaenoic acid	357.28	5.69	—	—	—	—	—	1.19	3.51	1.81	<0.001	<0.001	1.10	3.84	1.94	0.005	0.011
Tetradecanedioic acid	279.16	5.97	1.02	2.26	1.17	0.021	0.042	1.10	2.43	1.28	0.010	0.020	—	—	—	—	—
**Glucose metabolism**																	

Sedoheptulose	289.04	4.13	1.33	2.93	1.55	<0.001	<0.001	1.19	4.12	2.04	<0.001	<0.001	—	—	—	—	—
Malic acid	151.03	0.91	—	—	—	—	—	1.21	2.22	1.15	<0.001	0.002	1.11	2.95	1.56	0.003	0.007
Oxoglutaric acid	191.02	0.91	1.45	0.22	-2.16	<0.001	<0.001	1.21	0.18	-2.49	<0.001	<0.001	1.12	0.20	-2.35	0.001	0.003
Glucose 6-phosphate	259.03	4.51	1.00	0.10	-3.30	<0.001	<0.001	1.01	0.17	-2.57	0.001	0.003	—	—	—	—	—
L-Fucose 1-phosphate	243.03	4.34	1.45	0.04	-4.67	<0.001	0.002	1.18	0.07	-3.84	<0.001	0.003	—	—	—	—	—
Citric acid	288.99	1.20	1.42	0.14	-2.79	<0.001	<0.001	1.27	0.12	-3.03	<0.001	<0.001	1.16	0.10	-3.37	<0.001	0.002
Levoglucosan	179.06	0.91	—	—	—	—	—	—	—	—	—	—	1.05	0.78	-0.36	0.015	0.025
**Amino acid**																	
Aspartic Acid	132.05	3.69	1.55	1.11	0.15	<0.001	<0.001	1.44	0.37	-1.43	<0.001	<0.001	1.44	0.27	-1.91	<0.001	<0.001
D-Leucic acid	131.07	3.87	1.02	1.88	0.91	0.003	0.008	1.15	1.86	0.90	0.002	0.005	1.13	2.11	1.07	0.002	0.005
L-Histidine	156.08	0.78	1.36	1.46	0.55	<0.001	<0.001	1.31	1.81	0.86	<0.001	<0.001	1.20	1.79	0.84	0.007	0.014
DL-pipecolic acid	147.11	0.77	1.18	1.54	0.63	0.002	0.006	1.18	1.67	0.74	<0.001	0.002	1.18	1.76	0.81	0.007	0.015
L-Lysine	147.11	0.77	1.18	1.54	0.63	0.002	0.006	1.18	1.67	0.74	<0.001	0.002	1.18	1.76	0.81	0.007	0.015
Alanine	90.05	0.88	—	—	—	—	—	1.09	0.68	-0.55	<0.001	0.002	1.00	0.67	-0.58	0.007	0.015
L-Valine	118.09	0.92	—	—	—	—	—	1.19	0.70	-0.52	0.002	0.005	1.41	0.63	-0.67	<0.001	<0.001
3-Methylhippuric acid	194.08	3.83	—	—	—	—	—	—	—	—	—	—	1.38	0.56	-0.84	<0.001	<0.001
Ornithine	133.10	0.77	1.23	0.61	-0.72	<0.001	0.002	—	—	—	—	—	1.06	0.52	-0.94	0.005	0.010
Citrulline	174.09	0.87	1.19	0.58	-0.79	0.001	0.003	1.03	0.60	-0.74	0.015	0.026	1.02	0.51	-0.97	0.010	0.019
L-Arginine	175.12	0.79	1.20	0.67	-0.59	0.003	0.008	1.16	0.66	-0.60	<0.001	0.002	1.33	0.51	-0.98	<0.001	<0.001
L-Tryptophan	203.08	3.34	1.50	0.38	-1.40	<0.001	<0.001	1.43	0.37	-1.42	<0.001	<0.001	1.41	0.36	-1.46	<0.001	<0.001
Hippuric acid	178.05	3.69	1.62	0.34	-1.55	<0.001	<0.001	1.45	0.37	-1.43	<0.001	<0.001	1.46	0.28	-1.83	<0.001	<0.001
Homoarginine	189.13	0.86	1.40	0.38	-1.38	<0.001	0.001	1.32	0.40	-1.31	<0.001	0.002	1.39	0.27	-1.88	<0.001	<0.001
Uric acid	167.02	1.18	1.06	1.49	0.57	0.021	0.042	1.11	1.49	0.57	0.001	0.003	—	—	—	—	—
Kynurenine	207.10	5.40	1.42	0.94	-0.10	<0.001	<0.001	1.28	0.33	-1.62	<0.001	<0.001	—	—	—	—	—
L-Proline	116.07	0.98	1.06	0.78	-0.36	0.010	0.024	—	—	—	—	—	—	—	—	—	—
Phenylpyruvic acid	163.04	3.35	1.03	0.73	-0.45	0.010	0.024	0.98	0.73	-0.45	0.021	0.038	—	—	—	—	—

**Others**																	
L-Urobilinogen	595.35	5.65	1.22	280.43	8.13	<0.001	0.002	—	—	—	—	—	1.03	305.13	8.25	<0.001	0.002
L-Urobilin	593.33	4.14	1.16	164.01	7.36	<0.001	0.002	—	—	—	—	—	—	—	—	—	—
Xanthosine	283.08	3.42	1.27	21.33	4.41	0.005	0.013	1.30	33.03	5.05	<0.001	<0.001	1.37	11.97	3.58	<0.001	<0.001
Bilirubin	585.27	3.96	1.24	2.76	1.46	0.010	0.024	1.26	4.88	2.29	<0.001	<0.001	1.05	4.35	2.12	0.002	0.005
4-Methylindole	130.06	0.88	1.38	1.68	0.75	<0.001	0.001	1.07	1.49	0.58	0.003	0.007	—	—	—	—	—
Ubiquinone-1	249.11	5.60	1.17	0.94	-0.09	0.005	0.012	1.14	0.89	-0.18	<0.001	0.001	1.21	0.84	-0.25	0.001	0.003
Allantoic acid	177.06	0.79	1.00	0.70	-0.51	0.021	0.043	—	—	—	—	—	—	—	—	—	—
Xanthine	173.01	1.20	1.15	0.61	-0.71	0.003	0.008	1.31	0.40	-1.33	<0.001	0.002	1.05	0.50	-0.99	0.003	0.007
Dehydroascorbic acid	221.08	4.26	1.49	0.27	-1.88	<0.001	<0.001	1.35	0.24	-2.09	<0.001	<0.001	1.19	0.24	-2.05	0.001	0.003
Chenodeoxycholic acid 4-sulfate	471.24	5.06	—	—	—	—	—	1.08	3.55	1.83	<0.001	0.002	—	—	—	—	—
Deoxycholic acid	498.29	4.35	—	—	—	—	—	1.27	2.62	1.39	<0.001	0.001	—	—	—	—	—
Hypoxanthine	135.03	0.88	—	—	—	—	—	1.10	0.70	-0.52	0.003	0.007	—	—	—	—	—
Indole	162.06	3.40	—	—	—	—	—	1.04	1.58	0.66	0.003	0.006	—	—	—	—	—
Indolelactic acid	272.05	4.13	—	—	—	—	—	—	—	—	—	—	1.24	0.43	-1.21	0.001	0.003
L-Ascorbic acid	175.10	3.68	—	—	—	—	—	1.01	1.66	0.73	0.007	0.014	—	—	—	—	—
Taurine	124.01	0.86	—	—	—	—	—	1.13	2.20	1.14	<0.001	0.002	1.07	2.78	1.48	<0.001	<0.001

MZ, mass to charge ratio; VIP, variable importance in projection; FC, fold change, calculated as the mean value of the peak area obtained from the treatment group/mean value of the peak area obtained from the control group. FDR, false discovery rate.

Table S3 Ingredients and nutrient composition of the experimental diets for pregnant sheep (Ovis aries) in this study.

Items	Diets
Ingredient composition (% DM)	
Rye silage	42.3
Oat hay	34.6
Maize	12
Soybean	5.8
Barley	4.2
Premix1	1.1
Nutrient composition	
Metabolic energy (MJ/kg DM)	11.64
CP (% DM)	14.71
Crude fat (% DM)	2.95
NDF (% DM)	48.32
ADF (% DM)	29.09
Crude ash (% DM)	7.35
Calcium (% DM)	0.5
Phosphorus (% DM)	0.37

DM = dry matter basis.

[1]The premix (per kg) contained: vitamin A 64.8 mg, vitamin D2 1.35 mg, vitamin E 1080 mg, nicotinic acid 353 mg, Mn 537 mg, Cu 540 mg, Zn 2422 mg, Co 7.2 mg, I 32 mg, Se 18 mg, P 66 g, Ca 130 g, $NaHCO_3$ 89 g and NaCl 140 ~ 180 g.
